# *S*-Nitrosylation in endothelial cells contributes to tumor cell adhesion and extravasation during breast cancer metastasis

**DOI:** 10.1186/s40659-023-00461-2

**Published:** 2023-09-29

**Authors:** T. Koning, F. Cordova, G. Aguilar, J. Sarmiento, G. A. Mardones, M. Boric, M. Varas-Godoy, A. Lladser, W. N. Duran, P. Ehrenfeld, F. A. Sanchez

**Affiliations:** 1 Instituto de Inmunología, Facultad de Medicina, Universidad Austral de Chile, 511-0566 Valdivia, Chile; 2 Escuela de Graduados de Ciencias, Universidad Austral de Chile, 511-0566 Valdivia, Chile; 3 Instituto de Fisiología, Facultad de Medicina, Universidad Austral de Chile, 511-0566 Valdivia, Chile; 4 Departamento de Fisiología, Facultad de Ciencias Biológicas, Pontificia Universidad Católica de Chile, 8331150 Santiago, Chile; 5 Cancer Cell Biology Lab., Centro de Biología Celular y Biomedicina (CEBICEM), Facultad de Medicina y Ciencia, Universidad San Sebastián, 7510157 Santiago, Chile; 6 Centro Ciencia & Vida, Fundación Ciencia & Vida, 7780272 Santiago, Chile; 7 Facultad de Medicina y Ciencia, Universidad San Sebastián, Santiago, Chile; 8 Department of Pharmacology, Physiology and Neuroscience, New Jersey Medical School, Rutgers, The State University of New Jersey, Newark, NJ 07103 USA; 9 Instituto de Anatomía, Histología y Patología, Facultad de Medicina, Universidad Austral de Chile, 511-0566 Valdivia, Chile; 10 Centro Interdisciplinario de Estudios del Sistema Nervioso, Universidad Austral de Chile, 5110566 Valdivia, Chile; 11 Escuela de Medicina, Facultad de Medicina y Ciencia, Universidad San Sebastián, Valdivia, Chile

**Keywords:** Leukocyte adhesion, Nitric oxide, *S*-Nitrosylation, VCAM-1, Breast cancer metastasis

## Abstract

**Background:**

Nitric oxide is produced by different nitric oxide synthases isoforms. NO activates two signaling pathways, one dependent on soluble guanylate cyclase and protein kinase G, and other where NO post-translationally modifies proteins through *S*-nitrosylation, which is the modification induced by NO in free-thiol cysteines in proteins to form *S*-nitrosothiols. High levels of NO have been detected in blood of breast cancer patients and increased NOS activity has been detected in invasive breast tumors compared to benign or normal breast tissue, suggesting a positive correlation between NO biosynthesis, degree of malignancy and metastasis. During metastasis, the endothelium plays a key role allowing the adhesion of tumor cells, which is the first step in the extravasation process leading to metastasis. This step shares similarities with leukocyte adhesion to the endothelium, and it is plausible that it may also share some regulatory elements. The vascular cell adhesion molecule-1 (VCAM-1) expressed on the endothelial cell surface promotes interactions between the endothelium and tumor cells, as well as leukocytes. Data show that breast tumor cells adhere to areas in the vasculature where NO production is increased, however, the mechanisms involved are unknown.

**Results:**

We report that the stimulation of endothelial cells with interleukin-8, and conditioned medium from breast tumor cells activates the *S*-nitrosylation pathway in the endothelium to induce leukocyte adhesion and tumor cell extravasation by a mechanism that involves an increased VCAM-1 cell surface expression in endothelial cells. We identified VCAM-1 as an *S*-nitrosylation target during this process. The inhibition of NO signaling and *S*-nitrosylation blocked the transmigration of tumor cells through endothelial monolayers. Using an in vivo model, the number of lung metastases was inhibited in the presence of the *S*-nitrosylation inhibitor *N*-acetylcysteine (NAC), which was correlated with lower levels of *S*-nitrosylated VCAM-1 in the metastases.

**Conclusions:**

*S*-Nitrosylation in the endothelium activates pathways that enhance VCAM-1 surface localization to promote binding of leukocytes and extravasation of tumor cells leading to metastasis. NAC is positioned as an important tool that might be tested as a co-therapy against breast cancer metastasis.

## Background

Nitric oxide (NO) is a labile gas (approximate half-life of 5 s) that regulates different physiological functions in the organism [52]. It is produced by three different isoforms of nitric oxide synthases: endothelial (eNOS, mainly expressed in endothelium), inducible (iNOS, mainly expressed in the immune system) and neuronal (nNOS, expressed in the nervous system) [22]. NO produced by these isoforms exerts its functions through (a) the activation of soluble guanylate cyclase (GCs) or (b) the post-translational modification of proteins through a process called *S*-nitrosylation, which consists of the coupling of a NO molecule to free thiol groups of protein cysteine residues, forming an *S*-nitrosothiol. This post-translational modification regulates protein interactions, protein phosphorylation and protein traffic [27, 42, 48, 55].

Breast cancer is the second most common cancer in women (https://www.cancer.gov/types/breast). Increased NOS activity (eNOS, iNOS) and NO levels have been detected in metastatic breast tumors compared to benign or healthy breast tissue, suggesting a positive correlation between NO biosynthesis and degree of malignancy [47, 71, 79]. Moreover, the inhibition or genetic deletion of eNOS or iNOS in animal models of breast cancer reduces tumor growth and metastasis [26, 30]. Most communications show a positive role of NO and *S*-nitrosylation at the beginning of tumor formation and at the early stages of metastasis [61, 69], although other reports indicate an inhibitory role of *S*-nitrosylation [9, 45, 54]. This had led to the notion that NO plays a double-edged role in breast cancer [47]. Metastasis research has focused mainly on tumor cells and how *S*-nitrosylation of tumor proteins leads to the activation of early steps in the metastasis, such as epithelial to mesenchymal transition, migration and invasion [50, 69]. However, it is unknown whether later stages of metastasis, such as extravasation of tumor cells, where the endothelium plays a fundamental role, can be regulated by *S*-nitrosylation.

Endothelial cells regulate the binding and extravasation of cancer cells in the metastatic site [63, 70]. The attachment of cancer cells to the endothelium is the first step of the extravasation process. Cytokines and other factors produced by the primary tumor, circulating tumor cells and cells in the metastatic microenvironment promote binding between tumor cell integrins and endothelial adhesion molecules [51, 66, 80]. VCAM-1 in the endothelium participates actively in the metastatic process in different types of cancer, including breast cancer, by promoting adhesion of tumor cells to the endothelium, transmigration through endothelial monolayers and metastasis formation [34, 60, 65, 72, 78]. Alternatively, VCAM-1 in the endothelium also binds to monocytes and neutrophils that may bind tumor cells [37, 64, 66]. In fact, neutralizing VCAM-1 antibodies inhibit neutrophil and cancer cell infiltration and metastasis in in vivo models of breast cancer and melanoma [21, 78].

The fact that breast cancer cells adhere to sites of microcirculation that show an increase in NO production [83] suggest that the NO in the endothelium plays a key role during metastasis. The aim of this work was to investigate how eNOS-induced NO regulates endothelial function to promote metastasis. We demonstrated that IL-8 (a main cytokine produced by tumor cells) [20] and secreted factors from breast cancer cells activate NO signaling in the endothelium. The mechanism involves the increase in cell surface expression of VCAM-1 in the endothelium and VCAM-1-*S*-nitrosylation. Treatment with l-*N*ω-methylarginine (l-NMA, NOS inhibitor) and *N*-acetyl cysteine (NAC, an antioxidant that also inhibits *S*-nitrosylation) inhibited endothelial VCAM-1 cell surface expression, VCAM-1-*S*-nitrosylation, tumor cell transmigration through the endothelium and metastasis formation. Our results demonstrate that *S*-nitrosylation in the endothelium is an important regulator in the process of extravasation during metastasis and that tackling *S*-nitrosylation in the endothelium might be an important therapeutic strategy to control metastasis.

## Results

### IL-8 and conditioned medium from MDA-MB-231 cells induce leukocyte adhesion activating NO signaling

**Fig. 1:**
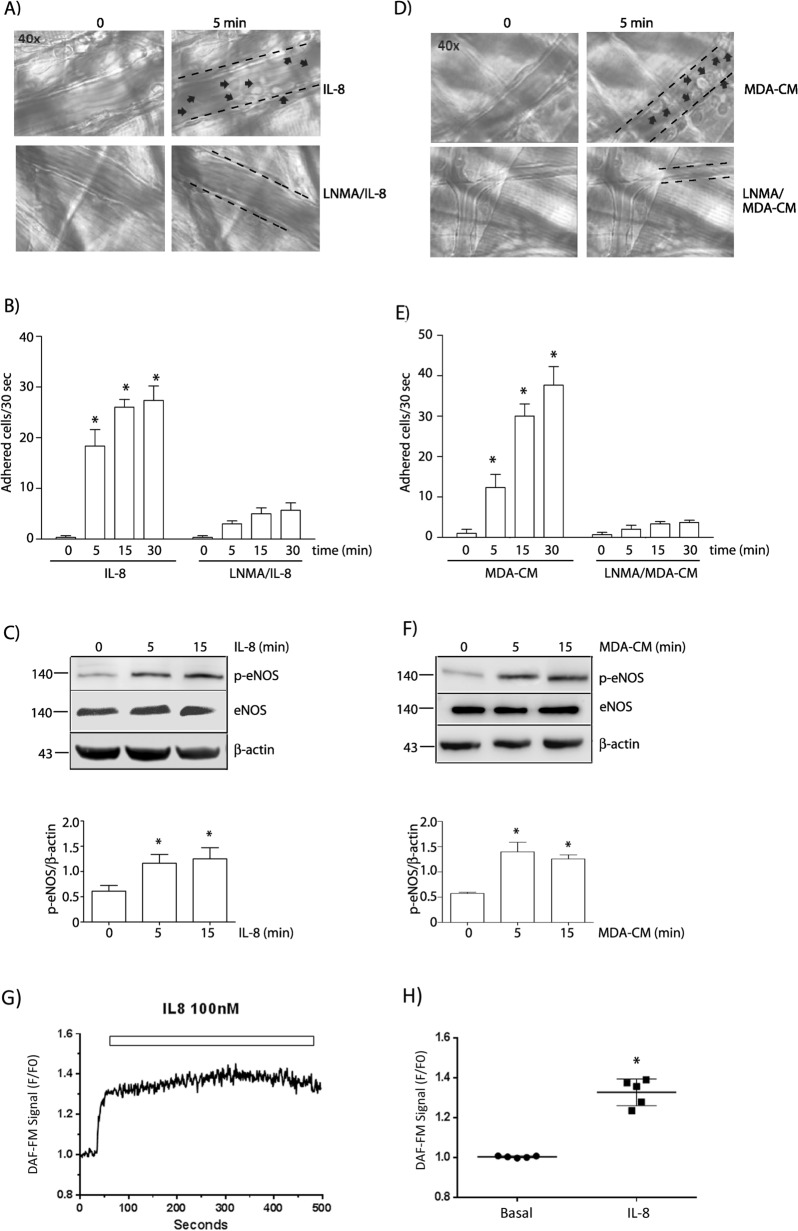
IL-8 and MDA-CM increase leukocyte adhesion through NO signaling. Cremaster muscle from Rockefeller mice was prepared for intravital microscopy. After stimulation with **A** IL-8 or **B** MDA-CM, leukocyte adhesion was recorded up to 30 min. Images show leukocyte adhesion at 5 min of stimulation with IL-8 or MDA-CM, respectively. Treatment with l-NMA before stimulation prevented leukocyte adhesion. Black arrows indicate leukocytes adhered to the endothelial cell surface delimited by dash lines. **C**, **D** Bar graphs showing statistical analysis of leukocyte adhesion to the cremaster stimulated with IL-8 and MDA-CM, respectively. Two-way ANOVA and Bonferroni’s Multiple Comparison Test, * p < 0.05 in comparison to time 0. n = 5. **E**, **F** Confluent EAhy926 cells were stimulated with IL-8 or MDA-CM for different periods of time and p-eNOS was detected by Western-blot. One-Way ANOVA and Bonferroni’s Multiple Comparison Test. * p < 0.05 in comparison to time 0; n = 5. **G** Representative image of real-time detection of NO induced by IL-8 in live EAHy926 cells. **H** Analysis of the increase in real-time detection of NO induced by IL-8 in live EAHy926 cells; paired t test, p < 0.0001; n = 5 independent experiments

The adhesion of tumor cells to the endothelium shares similarities with the adhesion of leukocytes to the endothelium, but the role of NO in these processes is unknown. For this reason, we first focused on testing whether NO signaling regulates leukocyte adhesion to the endothelium. We used the mouse cremaster muscle to study leukocyte adhesion in vivo by intravital microscopy. IL-8 and conditioned medium from MDA-MB-231 cells (MDA-CM) were applied topically to the cremaster muscle. IL-8 induced a significant increase in the number of leukocytes adhering to the endothelium (Fig. 1A, C). The inhibition of NO signaling with l-NMA before IL-8 application blocked this effect (Fig. 1A, C). Stimulation with MDA-CM also induced leukocyte adhesion to the endothelium, and it was also dependent on NO signaling (Fig. 1B, D). To demonstrate the involvement of eNOS-derived NO, we measured eNOS activation using eNOS phosphorylation at Ser 1177 in vitro as index of activation of the enzyme. IL-8 and MDA-CM induced eNOS phosphorylation at times corresponding to their effect on leukocyte adhesion (Fig. 1E, F). We also measured NO levels in response to IL-8 using DAF-FM. Figure 1G illustrates a representative result of one experiment showing the increase in the fluorescence signal for NO in the time. Figure 1H shows a significant increase in the accumulated fluorescence signal for NO after application of the agonist. These results demonstrate that IL-8 and MDA-CM induce leukocyte adhesion by activating the NO pathway.

### IL-8 and MDA-CM induce VCAM-1 expression at the cell surface depending on NO signaling

**Fig. 2:**
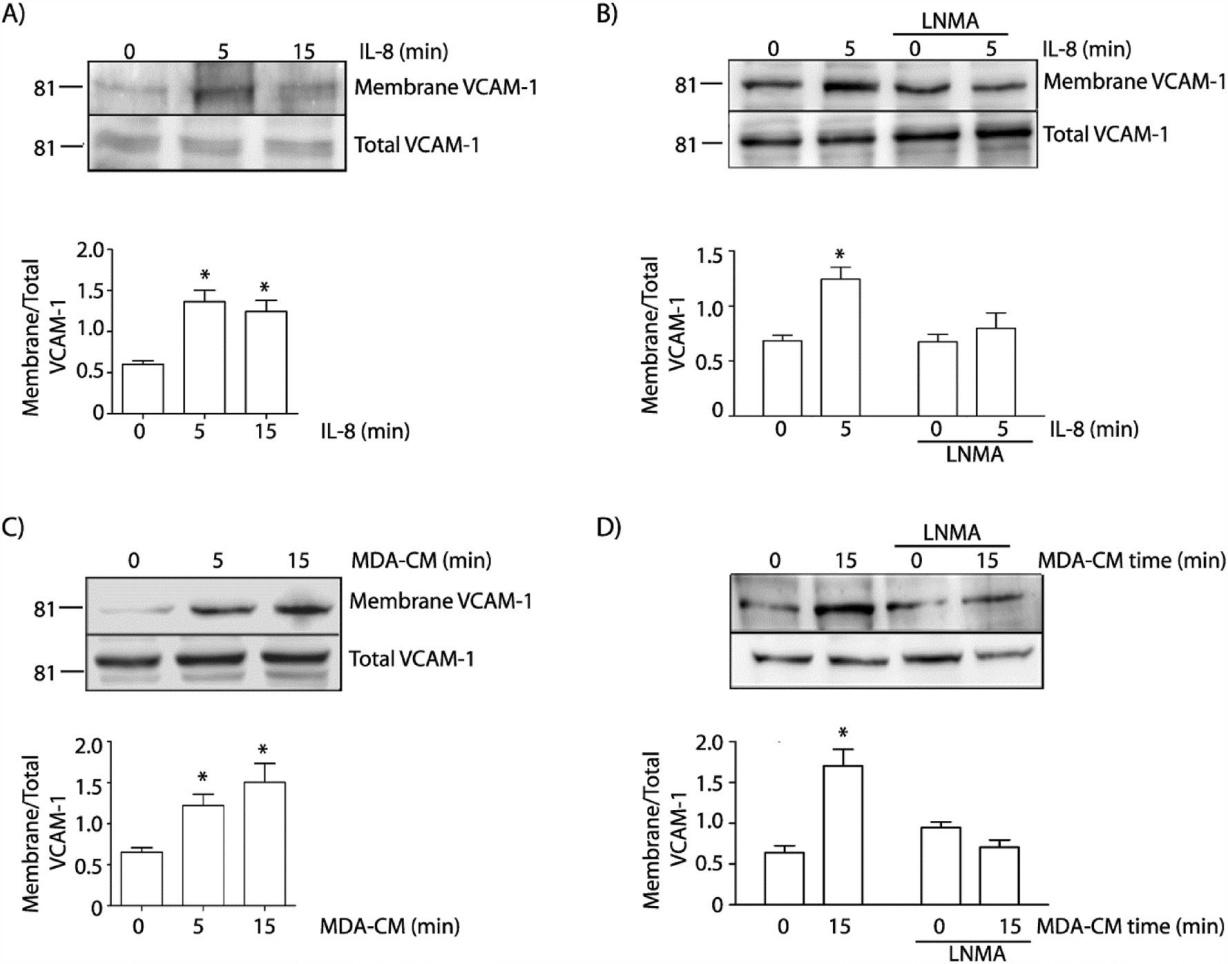
IL-8 and MDA-CM increase VCAM-1 endothelial cell surface expression through NO signaling. **A** EAhy926 monolayers were stimulated with IL-8 for 5 or 15 min and VCAM-1 in the cell surface was determined by cell surface biotinylation. IL-8 significantly increased VCAM-1 at the cell surface. One-Way ANOVA and Bonferroni’s Multiple Comparison Test, * p < 0.05 in comparison to time 0; n = 5. **B** Pretreatment of cell monolayers with l-NMA inhibited IL-8 stimulated VCAM-1 at the endothelial cell surface. Two-way ANOVA and Bonferroni’s Multiple Comparison Test, * p < 0.05; n = 5. **C** MDA-CM applied for 5 and 15 min significantly increased VCAM-1 at the cell surface. One-Way ANOVA and Bonferroni’s Multiple Comparison Test, * p < 0.05 in comparison to time 0; n = 5. **D** Pretreatment of cell monolayers with l-NMA inhibited MDA-CM induced VCAM-1 at the cell surface. Two-way ANOVA and Bonferroni’s Multiple Comparison Test, * p < 0.05; n = 3

VCAM-1 is an important protein implicated in tumor cell and leukocyte adhesion to endothelium [37, 59]. NO-mediated *S*-nitrosylation has been demonstrated to regulate protein traffic from and towards plasma membrane [32, 41, 42]. Thus, we investigated the effect of NO on VCAM-1 traffic to the cell surface, where its increase is directly related to the increase in leukocyte adhesion. Figure 2A shows that IL-8 induced a significant increase in the level of VCAM-1 at the endothelial cell surface as soon as 5 min of stimulation. This increase was independent of protein synthesis because total VCAM-1 levels were unchanged. The increase in VCAM-1 endothelial cell surface expression was dependent on NO signaling because it was inhibited in the presence of l-NMA (Fig. 2B). In the same way, MDA-CM also increased VCAM-1 endothelial cell surface expression (Fig. 2C), an effect that was inhibited by blocking NO signaling with l-NMA (Fig. 2D). These results demonstrate that IL-8 and MDA-CM induce VCAM-1 traffic to the endothelial plasma membrane activating the NO pathway.

### IL-8 and MDA-CM induce VCAM-1 S-nitrosylation

**Fig. 3:**
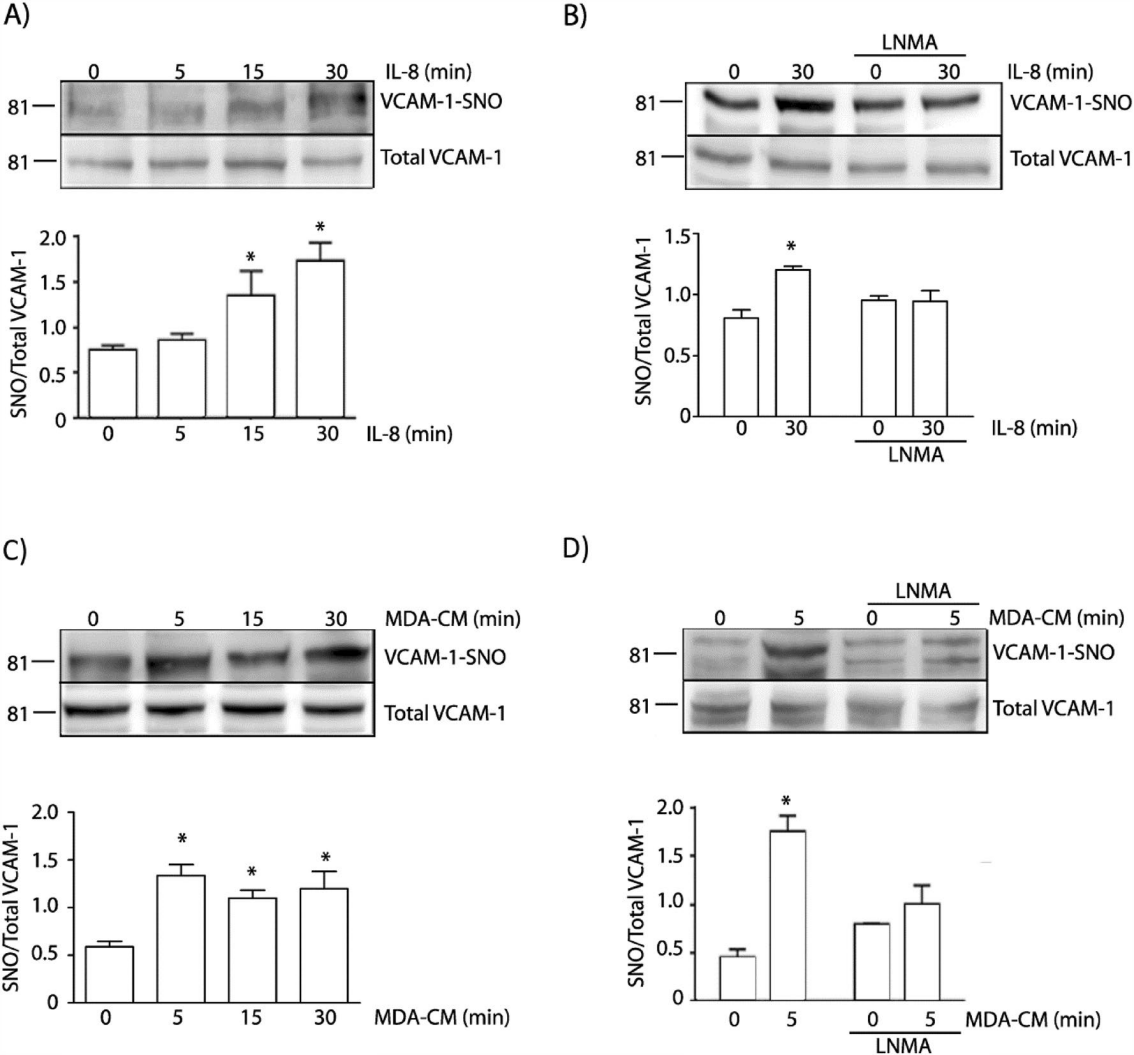
IL-8 and MDA-CM induce VCAM-1 *S*-nitrosylation depending on NO signaling. Confluent monolayers of EAhy926 cells were incubated with IL-8 (**A**) or MDA-CM (**C**) for 5 to 30 min. Cell lysates were obtained and processed by biotin switch to detect *S*-nitrosylated VCAM-1. Confluent monolayers of EAhy926 cells were incubated with IL-8 (**B**) or MDA-CM (**D**) in the presence or absence of l-NMA. Cell lysates were obtained and processed by biotin switch to detect-*S*-nitrosylated VCAM-1. For **A** and **C**, One-Way ANOVA and Bonferroni’s Multiple Comparison Test, * p < 0.05; n = 5. For **B** and **D** Two-way ANOVA and Bonferroni’s Multiple Comparison Test, * p < 0.05; n = 5

Because protein *S*-nitrosylation correlates with changes in protein localization [27, 32, 42], we tested whether or not VCAM-1 is *S*-nitrosylated by IL-8 or MDA-CM stimulation. Figure 3A shows that stimulation of EAhy926 cells with IL-8 induced VCAM-1 *S*-nitrosylation with stronger effect at 30 min of treatment, which was inhibited in the presence of l-NMA (Fig. 3B). MDA-CM also induced VCAM-1 *S*-nitrosylation with stronger effect at 5 min of treatment, which was inhibited by l-NMA (Fig. 3C, D). These results demonstrate that the levels of VCAM-1-*S*-nitrosylation stimulated by IL-8 and MDA-CM correlate with the increase in their expression at the cell surface.

### *S*-nitrosylation regulates breast cancer metastasis

**Fig. 4:**
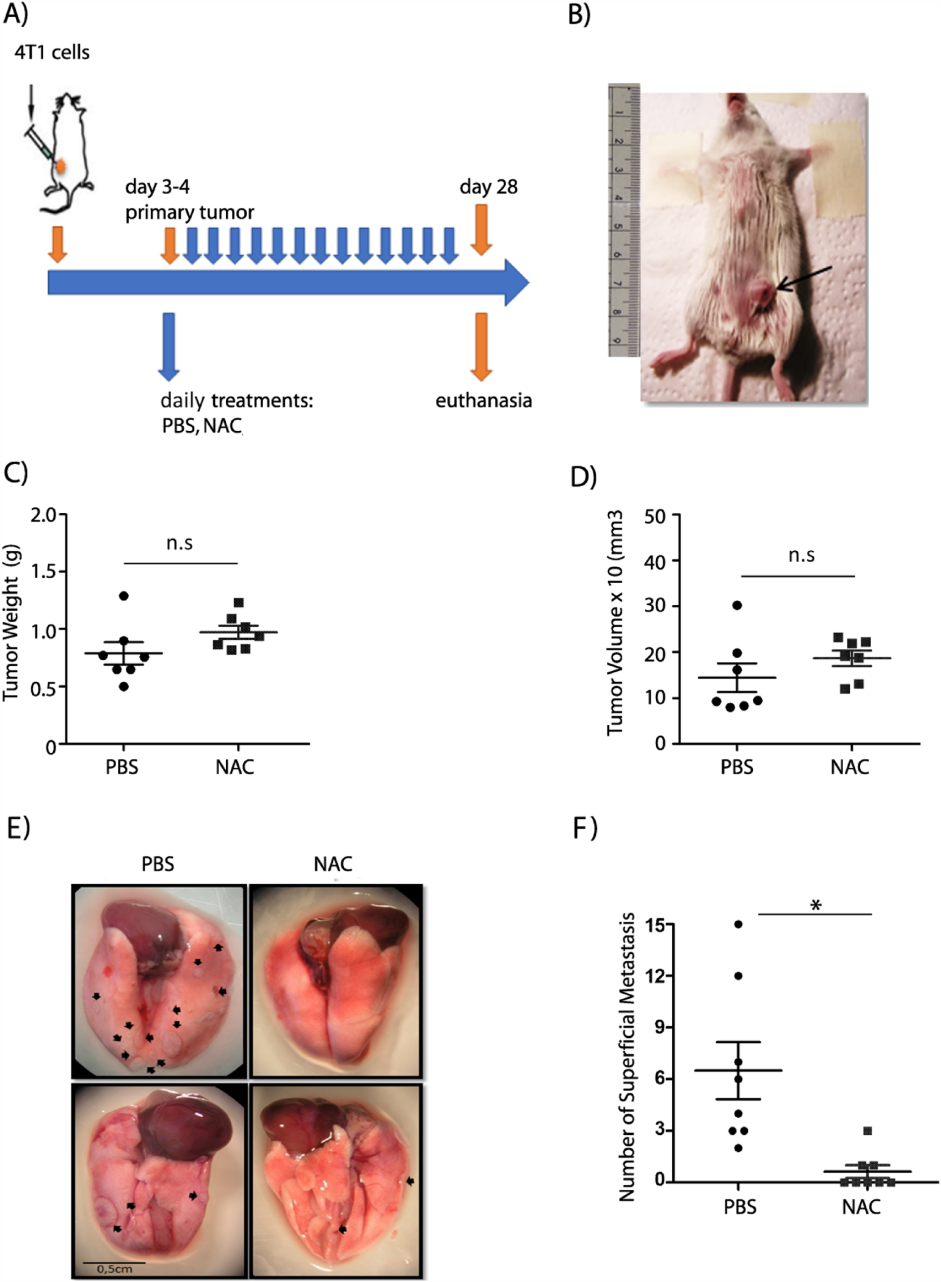
*S*-Nitrosylation regulates lung metastasis in vivo. **A** Scheme of the treatments conducted in the murine syngeneic model of breast cancer. **B** Breast tumors were generated in BALB-C female mice by 4T1 cell injection in the fourth mammary pair. Representative image of the tumor formed after four weeks post-injection of 4T1 cells. Slices. **C** Bar graph showing the quantification of tumor weight. T-test; n.s = non-significant. **D** Bar graph showing the quantification of tumor volume. One-Way ANOVA and Bonferroni’s Multiple Comparison Test, * p < 0.05; n = 7. **E** Representative images of lungs post-euthanasia. Black arrows indicate surface metastases. **F** Graph showing the number of superficial metastases counted in each study group. One-Way ANOVA and Bonferroni’s Multiple Comparison Test, * p < 0.05; n = 7

The experiments described above suggest that NO signaling regulates leukocyte adhesion by increasing VCAM-1 in the endothelial cell surface. The same mechanism might occur when circulating tumor cells colonize secondary sites in the extravasation process. To test that, we used the 4T1 mouse model of metastatic breast cancer, since 4T1 cells have the same metastatic characteristics as MDA-MB-231 cells. In these in vivo experiments, we used NAC, an antioxidant that inhibits *S*-nitrosylation [42, 68], instead of l-NMA that would inhibit all NOS and could generate systemic physiological damage. Female BALB-C mice were injected with 4T1 cells into the mammary fat pad. After primary tumor formation, mice were separated in two groups: control and NAC treated (Fig. 4A). Immunohistochemical analysis showed that breast tumors were correctly formed (Fig. 4B). NAC treatment did not influence the final size and volume of primary tumor (Fig. 4C, D). However, there was a significant reduction in the number of superficial metastases observed in the group treated with NAC compared to the control group (Fig. 4E, F).

### Lung metastasis has increased levels of *S*-nitrosylation and VCAM-1-*S*-nitrosylation

**Fig. 5:**
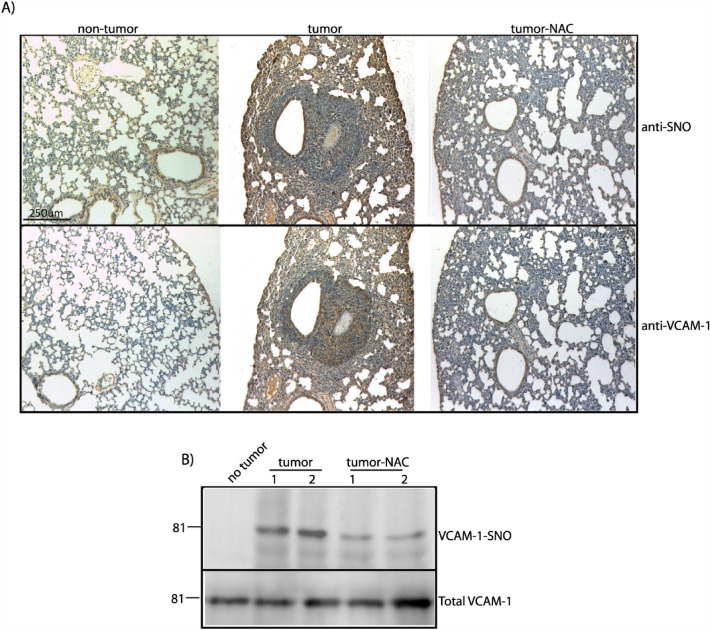
VCAM-1 and VCAM-1-*S*-nitrosylation in lung metastasis. Breast tumors were generated in BALB-C female mice, the lungs were extracted after 28 days and processed to detect: **A** *S*-nitrosylation (upper panel) and VCAM-1 (lower panel) expression in the non-tumor and tumor tissue by immunohistochemistry. n = 5. **B** *S*-nitrosylated VCAM-1 in lung tissues detected by biotin switch. n = 2

Immunohistochemical analyses of lung metastasis showed high levels of *S*-nitrosylated proteins in lung tumors compared to non-tumor lung (Fig. 5A, upper panel). Treatment with NAC diminished the levels of *S*-nitrosylated proteins (Fig. 5A). Similarly, VCAM-1 levels were increased in lung metastasis compared to normal tissue (Fig. 5A, lower panel), and NAC treatment diminished VCAM-1 levels in lung metastasis. To test whether VCAM-1 is *S*-nitrosylated in lung metastasis, we performed a biotin switch analysis. Figure 5B shows higher levels of *S*-nitrosylated VCAM-1 in lungs from tumors compared to lung from control mice. NAC treatment inhibited VCAM-1-*S*-nitrosylation in metastasis (Fig. 5B). These results indicate that treatment with inhibitors of *S*-nitrosylation inhibit the levels of *S*-nitrosylated VCAM-1 in metastasis, which correlated with lower metastasis formation.

### Effect of NAC on metastasis is due to effects on endothelial cells

**Fig. 6:**
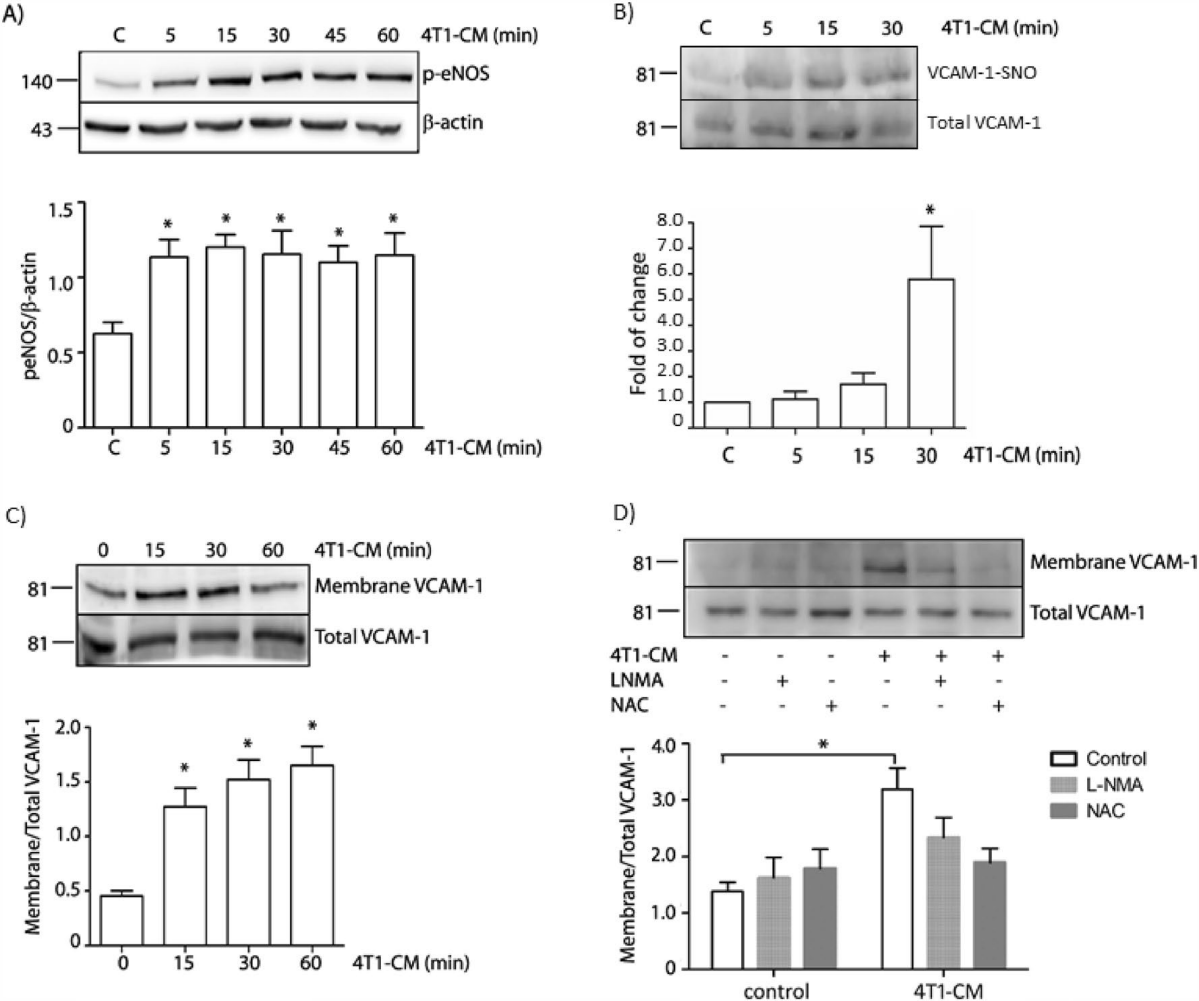
4T1-CM increases VCAM-1 cell surface expression by activating the eNOS-*S*-nitrosylation pathway. Confluent monolayers of EAhy926 cells were treated with 4T1-CM for different periods of time and processed to detect: **A** p-eNOS by Western-blot. One-Way ANOVA and Bonferroni’s Multiple Comparison Test, * p < 0.05; n = 5. **B** VCAM-1-*S*-nitrosylation by biotin switch. One-Way ANOVA and Bonferroni’s Multiple Comparison Test, * p < 0.05; n = 4. **C** Membrane VCAM-1 by cell surface biotinylation. One-Way ANOVA and Bonferroni’s Multiple Comparison Test, * p < 0.05; n = 4. **D** Membrane VCAM-1 in the presence of l-NMA and NAC. Two-Way ANOVA and Bonferroni’s Multiple Comparison Test. * p < 0.05 in comparison to non-treatment; n = 3

To corroborate that the effect on metastasis is due to an effect on endothelial cells, we explored the effect of conditioned medium from 4T1 cells (4T1-CM) on endothelial cells. We found that stimulation of endothelial cells with 4T1-CM induces eNOS activation, VCAM-1 *S*-nitrosylation and VCAM-1 expression at the cell surface (Fig. 6A–C). VCAM-1 expression at the cell surface was inhibited in the presence of NAC and l-NMA (Fig. 6D). These results suggest that the effects of NAC seen in metastasis might be due to a diminished VCAM-1 expression at the endothelial cell surface regulated by *S*-nitrosylation.

### NO-mediated *S*-nitrosylation regulates tumor cell extravasation through endothelial monolayers

**Fig. 7:**
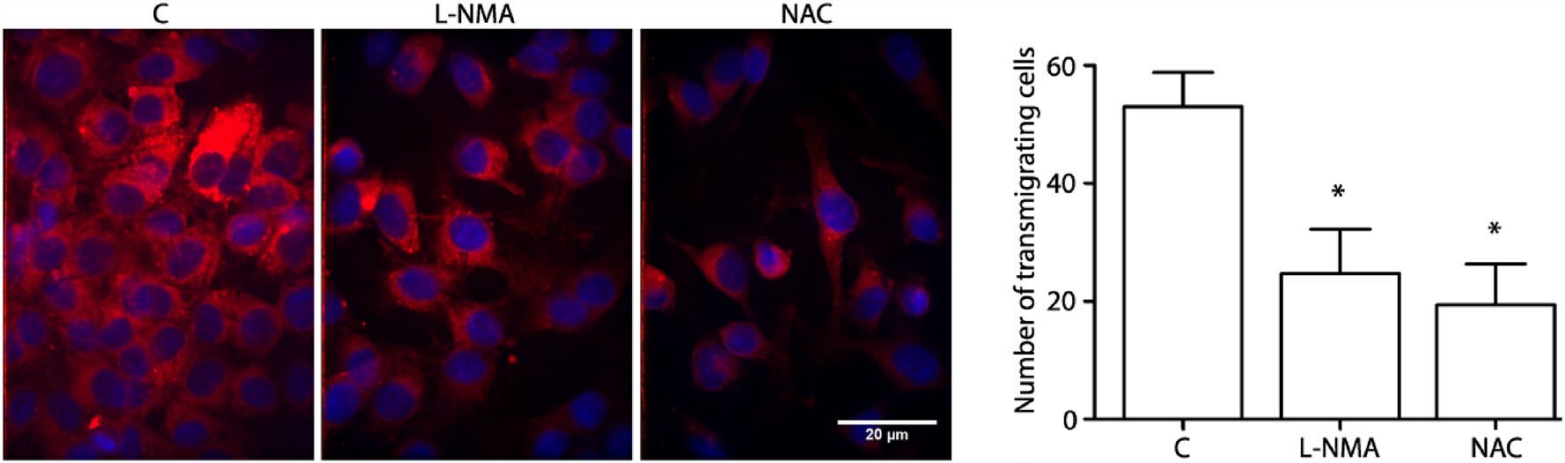
l-NMA and NAC inhibit tumor cell transmigration. MDA-mCherry cells were added to EAhy926 monolayers growing in Transwell filters and previously incubated with 300 µM l-NMA for 1 h or 2.5 mM NAC for 1.5 h. Transmigration was allowed for 6 h. Transwell inserts were removed, fixed, and mounted. MDA-mCherry cells were counted on the basal side of the transwell in random fields. T test, * p < 0.05 in comparison to control (C:transmigrating cells with any compound); n = 4

VCAM-1 expression is highly correlated with tumor cell adhesion and extravasation [37, 59]. To test the role of *S*-nitrosylation in tumor cell extravasation, we carried out transmigration assays by incubating EAhy926 endothelial cells with breast tumor cells MDA-MB-231 stably expressing the fluorescent protein mCherry (MDA-mCherry). To determine the role of NO and *S*-nitrosylation in the extravasation of tumor cells, we used l-NMA and NAC. The analysis revealed a significant number of MDA-mCherry cells transmigrating through the endothelial monolayers (Fig. 7). Inhibition with l-NMA or NAC blocked tumor cell transmigration. These data indicate that tumor transmigration is regulated by *S*-nitrosylation.

## Discussion

Tumor cell adhesion to endothelial cells is the first step in the extravasation of tumor cells to the metastatic site. This adhesion is mediated directly by binding of tumor cells to adhesion proteins in the endothelium or indirectly by binding to leukocytes that bind to the endothelium using the same adhesion proteins. In this research, we demonstrate that IL-8 and conditioned medium from triple negative breast cancer cells induce leukocyte adhesion and increase the levels of VCAM-1 at the cell surface. These changes were dependent on the activation of the NO pathway and correlated with VCAM-1-*S*-nitrosylation. Furthermore, inhibition of *S*-nitrosylation in vivo*,* using NAC treatment, inhibited the development of metastasis associated with diminished levels of VCAM-1 *S*-nitrosylation in the metastasis. The inhibition of the *S*-nitrosylation pathway also inhibited the transmigration of tumor cells through the endothelium.

Our results demonstrate that NO increases the levels of VCAM-1 at the cell surface. In a physiological situation, the stimulation of NO production may occur by the release of agents produced by the primary tumor, circulating tumor cells and cells in the metastatic microenvironment. Pro-inflammatory cytokines secreted by breast tumor cells might bind to receptors in endothelial cells leading to the activation of eNOS signaling. In fact, we have demonstrated that the stimulation of endothelial cells with different agonists secreted by tumor cells and elevated in serum of breast cancer patients activate eNOS and the *S*-nitrosylation pathway in the endothelium resulting in loss of endothelial integrity [20, 42, 82]. In addition, we have previously demonstrated that blocking of IL-8 receptor CXCR2 or depletion of Gal-8 in tumor cells inhibits NO signaling mediated by conditioned medium from glioblastoma and breast tumor cells [28, 82]. A similar mechanism might be activated by IL-8 binding to its receptor in endothelial cells that causes NO signaling, leading to VCAM-1 *S*-nitrosylation and localization in the plasma membrane to bind circulating tumor cells. Furthermore, the physical trapping of tumor cells in small blood vessels [83] can increase the shear stress in the vessel wall stimulating NO production, and at the same time favor the local secretion of agents from circulating tumor cells that stimulate NO production, which can cause an increased VCAM-1 cell surface expression on endothelial cells to bind tumor cells.

VCAM-1 expression has been described in several types of cancer including breast cancer [34, 60, 65, 72, 78]. The administration of neutralizing VCAM-1 antibodies impairs breast cancer cell adhesion to endothelial cells, transmigration through endothelial monolayers and metastasis formation [56, 60, 65]. Alternatively, VCAM-1 in the endothelium also binds to monocytes and neutrophils [76] that may bind tumor cells. In fact, inhibition of PMNs infiltration can be achieved by pretreating PMNs with soluble vascular cell adhesion molecule-1 [14]. In addition, neutralizing VCAM-1 antibodies inhibit neutrophil and cancer cell infiltration and metastasis in in vivo models of breast cancer [21, 78]. Here, we demonstrate that increased VCAM-1 expression in the endothelial cell surface is mediated by NO signaling and correlated with VCAM-1 *S*-nitrosylation. NO and *S*-nitrosylation have been demonstrated to regulate protein traffic toward and from the plasma membrane [32, 42]. In endothelial cells the treatment with a NO scavenger for a relative short period of time (24 h) inhibits the secretion of recombinant horse radish peroxidase (HRP), but sustained treatment improves secretion [40]. In neurons, NSF-*S*-nitrosylation increases α-amino-3-hydroxy-5-methyl-4-isoxazolepropionic acid (AMPA) receptors traffic to the plasma membrane [31]. In contrast, in endothelial cells, NSF-*S*-nitrosylation inhibits the translocation of Weibel-Palade bodies containing P-selectin to the plasma membrane [44]. Since we demonstrated that VCAM-1 itself is *S*-nitrosylated, it is possible that this *S*-nitrosylation contributes to VCAM-1 traffic to the plasma membrane. Very little is known about the transport route of VCAM-1 to the cell surface. Some reports have indicated that VCAM-1 cell surface expression requires its association to a family of proteins called tetraspanins CD151 or CD9 [13], which regulate biosynthetic maturation and traffic of their associated proteins [8]. Interaction of tetraspanins with partner proteins occurs in the *trans*-Golgi network and facilitates their exit and transport to the plasma membrane [8]. Studies have suggested that adhesion proteins (VCAM-1 and ICAM-1) co-cluster with tetraspanins through their transmembrane domains to promote their cell surface expression [5, 6]. Interestingly, cysteine 668 is located in the transmembrane domain of VCAM-1. *S*-nitrosylation of this residue might contribute to the interaction of VCAM-1 and tetraspanins to improve its transport to the cell surface. Interestingly, we observe that when we stimulate with IL-8, *S*-nitrosylation occurs later than when we stimulated with MDA-MC. Furthermore, in the case of IL-8, *S*-nitrosylation occurs at times later than the arrival of VCAM-1 at the membrane. It could be that the initial arrival of VCAM-1 at the membrane depends on NO-regulated mechanisms different than VCAM-1 *S*-nitrosylation and subsequent *S*-nitrosylation of this protein could stabilize it on the membrane.

We also demonstrated that *S*-nitrosylation regulates the transmigration of tumor cells through endothelial monolayers. This may result from an improved adhesion of tumor cells to the endothelium. In fact, preceding reports have indicated that NO enhanced fibrosarcoma and squamous carcinoma cell adhesion to the endothelium [58, 81]; breast tumor cells adhered to sites of microcirculation that show an increase in NO production [83] and NOS inhibitors blocked the adhesion and transmigration of small cell lung carcinoma and breast tumor cell to the endothelium [19, 75] pointing out a predominant role of NO in cancer cell adhesion to the endothelium; however, the mechanism (sGC-PKG or *S*-nitrosylation) or the actors involved are unknown. The effect of *S*-nitrosylation on transmigration of tumor cells may also be due to a direct effect on the endothelial barrier. We have previously demonstrated that conditioned medium from tumoral cells and agents secreted by tumor cells (i.e., Gal-8 and IL-8) induce *S*-nitrosylation of adherens junction proteins destabilizing the endothelial barrier [28, 82], which might facilitate the passage of tumor cells.

Research in breast cancer has focused mainly on tumor cells, where it has been demonstrated that *S*-nitrosylation of tumor proteins leads to the activation of early steps in the metastasis, such as epithelial to mesenchymal transition, migration and invasion [11, 18, 25, 50, 53, 67–69, 77]. However, it is unknown whether later stages of metastasis, such as extravasation of tumor cells, where the endothelium plays a fundamental role, may be regulated by *S*-nitrosylation. Ours is the first report demonstrating that *S*-nitrosylation in the endothelium might regulate metastasis. This is important because tackling specifically *S*-nitrosylation offers the advantage to avoid collateral effects such as hypertension induced by general NOS inhibitors.

Our results showing NO as a signal that promotes cell adhesion to the endothelium are in strong contrast to studies showing an inhibitory role of NO in this process [16, 24, 38, 73]. Besides the differences in the cell and animal models used, we propose that these discrepancies can be explained by the fact that the physiological effects of NO strictly depend on its concentration, duration of exposure, location, and activity of NOS isoforms [2, 3]. In non-stimulated cells, basal NO production could maintain an anti-adhesive phenotype [23, 44]. A slight increase in NO, presumably induced by eNOS, can promote an adhesive phenotype in endothelial cells [35, 49]. On the other hand, NO produced at higher concentration by iNOS stimulation inhibits adhesion due to *S*-nitrosylation of NFKB, which blocks its entry to the nucleus leading to inhibition of adhesion protein synthesis [43].

In vivo, in the model of metastatic breast cancer, our results demonstrate increased levels of *S*-nitrosylation in the metastasis. Specifically, VCAM-1-*S*-nitrosylation was increased in lung metastasis. NAC treatment inhibited metastasis, total *S*-nitrosylation and VCAM-1 *S*-nitrosylation in lung tissue. NAC is a widely known antioxidant, approved by the FDA in the treatment of chronic obstructive lung disease and as antidote to acetaminophen overdose [10, 46]. Because NAC use is already approved by the FDA, the repositioning of this drug to treat breast cancer metastasis might be easily reached. In fact, NAC has been clinically tested as a chemo-preventive agent in USA and Europe [1, 57]. Several studies in vitro and in vivo have demonstrated an anti-carcinogenic and anti-metastatic effect of NAC [1, 15, 17]. In contrast, other studies have shown no benefit of NAC in the development of tumors and metastasis [57, 74], and even an increased metastasis was observed after NAC treatment in estrogen receptor-positive breast cancer [57]. Therefore, the anti-metastatic properties of NAC are not clear. Furthermore, these studies have been performed applying NAC at the beginning of tumor formation and it is unknown if it can act after the primary tumor is formed to specifically stop the metastasis. Considering that breast cancer detection occurs after the primary tumor is formed and generally has already advanced, in our study we applied NAC after primary tumor formation as it would be applied in a possible treatment for cancer patients. NAC effects have been attributed to inhibition of functions in tumor cells such as reduced expression of vimentin, inhibition of EMT, inhibition of cell migration, invasion, and gelatinase production [4, 62]. Our in vitro experiments demonstrated that NAC treatment also affect endothelial function reducing tumor cell transmigration through endothelial monolayers, VCAM-1 traffic to the plasma membrane and VCAM-1 *S*-nitrosylation. Our results coincide with other studies showing that NAC inhibits the transmigration of MDA-MB-231 cells through HUVEC monolayers, disruption of the adherens junction and VE-cadherin phosphorylation induced by interaction of MDA-MB-231 cells with HUVECs [29]. Furthermore, we previously demonstrated that NAC inhibits the disruption in the endothelial barrier in response to pro-inflammatory agents by inhibiting VE-cadherin phosphorylation and *S*-nitrosylation of β-catenin, p120 and VE-cadherin [27, 42]. These data together indicate that NAC effects on metastasis are not only due to effects in tumor cells but also in endothelial cells. Our results have important implications in the process of extravasation of tumor cells at late stages of the metastasis and could be the basis for the development of new therapies using NOS inhibitors or NAC in combination with other drugs as therapeutic strategy to prevent metastasis in breast cancer. In fact, the possible application of l-NMA in combination with other drugs is already beginning to be addressed in clinical trials [12, 39], however, the mechanism is unknown. Our results might also be extrapolated to other types of cancer that cause metastasis.

## Conclusions

**Fig. 8:**
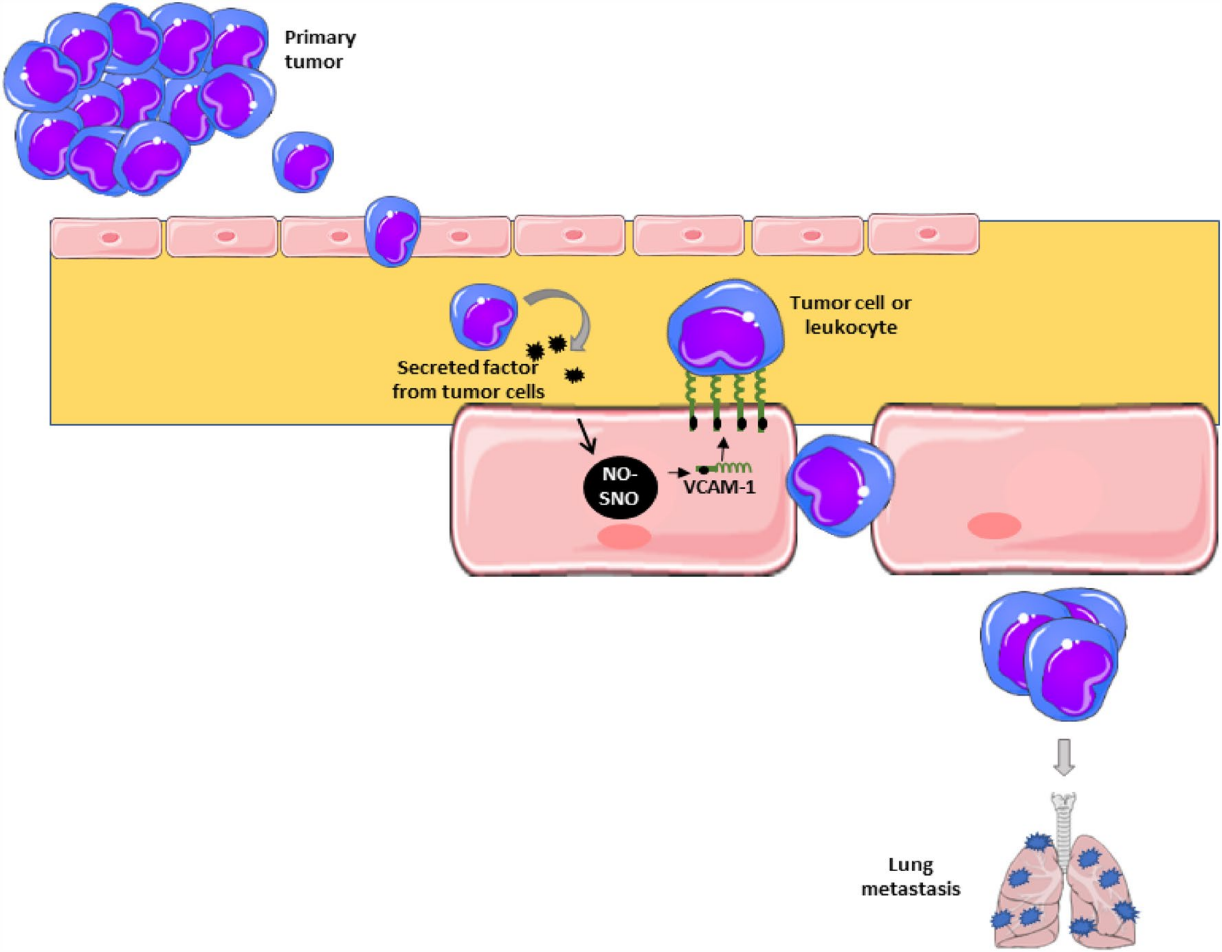
Secreted factors from tumor cells can activate the *S*-nitrosylation pathway in the endothelium to promote VCAM-1 expression in the cell surface leading to binding of tumor cell or leukocyte to promote extravasation and posterior development of metastasis

Overall, our data indicate that *S*-nitrosylation in the endothelium activates pathways that enhance surface localization of VCAM-1 to promote binding of leukocyte and/or tumor cells and extravasation leading to metastasis. VCAM-1-*S*-nitrosylation might be considered as a marker of cancer progression. Furthermore, NAC, a drug approved by the FDA for the treatment of other diseases, is positioned as an important tool that could be tested as a co-therapy against breast cancer metastasis (Fig. 8).

## Methods

### Reagents

Recombinant IL-8 (Joseph et al. 36) was used at 100 nM in in vitro experiments and at 1 µM in in vivo experiments. *NG*-methyl-l-arginine (l-NMA) (Sigma Chemicals, St. Louis, MO) and *N*-acetyl-l-cysteine (NAC) (Calbiochem, San Diego, CA). l-NMA (150 µM) and NAC (2.5 mM) were added 1 h and 1.5 h before agonist application, respectively. Administration of chemicals was continued throughout the experiment.

### Antibodies

Mouse anti-VCAM-1 (Abcam). Mouse anti-peNOS-Ser1177 (BD Transduction Laboratories), mouse anti-β-actin and rabbit anti-*S*-Nitroso-Cysteine (Cys-NO) (Sigma).

### Cell culture and conditioned medium

Immortalized human venous endothelial cells, EAhy926 (ATCC® CRL2922™), were grown in Dulbecco’s modified Eagle’s medium (DMEM) 10% FBS and supplemented with 2 mM l-glutamine, 100 U/ml penicillin, 100 µg/ml streptomycin, 2.5 μg/ml fungizone and 100 μM sodium hypoxanthine, 0.4 μM aminopterin, and 16 μM thymidine. Human mammary adenocarcinoma cell line MDA-MB-231 (ATCC® HTB-26™) were grown in DMEM-F12 10% FBS and supplemented with 100 U/ml penicillin, 100 µg/ml streptomycin, 2.5 µg/ml fungizone. We generated MDA-MB-231 cells stably expressing the mCherry member of monomeric red fluorescent proteins (MDA-mCherry) by transfecting cells with the pmCherry-C1 mammalian expression vector (Clontech) and expanding a one-cell colony selected with 1 mg/ml Geneticin (G418) (Calbiochem). MDA-mCherry cells were grown as the parental cells, but additionally supplemented with 1 mg/ml G418. Murine breast cancer cell line 4T1 (ATCC CRL-253) was grown in RPMI 10% FBS and supplemented with 100 U/ml penicillin, 100 µg/ml streptomycin, 2.5 μg/ml fungizone. Conditioned medium from MDA-MB-231 and 4T1 cells (MDA-CM and 4T1-CM) was obtained from 80% confluent cells growing for 48 h in medium without serum. The medium was decanted and cleared by centrifugation at 1075×*g* for 5 min and stored at − 80 °C until use.

### Western blot analysis

Confluent cells growing in a 100- or 35-mm plate were serum starved (2% FBS) overnight or 3 h minimum. IL-8, MDA-CM or 4T1-CM was applied to the cells for different periods of time. Cells were washed twice with ice cold PBS and scraped in 600 µL (100 mm plate) or 100 µl (35 mm plates) lysis buffer (50 mM Tris, 150 mM NaCl, 0.1 mM EDTA, 0.1 mM EGTA, 1% Triton X-100 and protease inhibitor mixture) and incubated in ice with shaking for 30 min. Lysates were obtained by centrifugation at 10,000×*g* for 15 min at 4 °C. Proteins were separated by SDS-PAGE and blotted onto PVDF membranes for detection with specific antibodies (anti-VCAM-1 1:750, anti p-eNOS 1:2000 and anti-ß-actin 1:5000). Proteins of interest were detected by ECL (Pierce). We analyzed Western blots quantitatively using the NIH ImageJ program.

### Real-time detection of NO

We use a modification of the protocol previously described by us [2]. Briefly, confluent EAHy926 cells were loaded with DAF-FM Diacetate (5 mM, Invitrogen, Cat. No. D-23844) and examined for increases in NO following addition of IL-8 100 nM. For the real-time detection of NO, a spinning disk confocal system (CSU-X1, Yokogawa) equipped with a DMi8 inverted microscope, an oil immersed 63 × 1.4 NA objective (Leica Biosystems) and temperature (37 °C) and humidity control system was used (Chamlide TC Live Cell Imaging (LCI). To minimize exposure time, resolution was reduced at 2 × 2 binning on a CCD-based (EMCCD iXon Life 888, ANDOR, Oxford Instruments) and the excitation laser line of 488 nm (20w) was attenuated to 2%. Exposure times were 50 ms and multiple exposures over time were collected at one image per second for 500 s. The imaging sequence was initiated, and 30 s later the stimulus was added. For the analysis, 9 individual regions of interest (ROI) were analyzed for each plate using the software SlideBook 6 (Intelligent Imaging Innovations (3i). Data are expressed as the mean fluorescence intensity (F) relative to the mean fluorescence intensity before stimulus application (F0). This value is expressed as F/F0.

### Cell surface biotinylation

Confluent cells growing in a 100-mm plate were serum starved (2% FBS) overnight or 3 h minimum. Endothelial cells were stimulated with IL-8 and conditioned medium from breast cancer cell lines for different periods of time. Endothelial cells were washed twice with PBS and incubated with 1 mL of EZ-Link Sulfo-NHS-Biotin (Pierce) for 30 min at 4 °C. Endothelial monolayers were washed 3 times with Tris buffered saline, scraped in 600 µl lysis buffer (50 mM Tris, 150 mM NaCl, 0.1 mM EDTA, 0.1 mM EGTA, 1% Triton X-100, protease inhibitor mixture), and incubated on ice with shaking for 30 min. Precleared lysates were obtained by centrifugation at 10,000×*g* for 5 min at 4 °C, and incubated afterward for 2 h at 4 °C with 50 µl of NeutrAvidin-coated agarose beads (Pierce). Beads were collected by centrifugation at 14,000×*g* and washed 6 times with lysis buffer. The beads with biotinylated proteins were suspended in loading buffer and proteins were separated by SDS-PAGE. Separated proteins were blotted onto PVDF membranes and detected with antibodies specific for VCAM-1.

### Biotin-switch assay

One hundred µg of total protein obtained from cellular lysates were denatured with sodium dodecyl sulfate (SDS) in the presence of methyl methanethiosulfonate (MMTS) [33]. After acetone precipitation to remove excess MMTS, 1 mM ascorbate and 4 mM biotin-HPDP (*N*-[6-(biotinamido hexyl]-3′-(2′-pyridyldithio propionamide) were added to reduce the S–NO bond and label the reduced thiol with biotin, respectively. Biotinylated proteins were recovered and analyzed according to our previous publications [2, 28]. To analyze VCAM-1 *S*-nitrosylation in the metastasis, lung tissue was extracted in lysis buffer and processed by biotin switch.

### Extravasation assay

1 × 10^5^EAhy926 cells were grown to confluence onto gelatin coated Transwell inserts (8 µm pore size). 2 × 10^4^ MDA-MB-231-mCherry cells in DMEM 1% FBS were added to the upper compartment of the chamber and DMEM 10% FBS was added to the lower compartment of the chamber. After incubation at 37 °C for 6 h, the top side of the insert membrane was scrubbed with a cotton swab and the bottom side was fixed with 4% paraformaldehyde to count tumor cells in 5 random visual fields and photographed under a fluorescence microscope. In the experiments using l-NMA and NAC, these inhibitors were added before tumor cells and maintained throughout the experiment.

### In vivo experiments

Protocols for animal experiments were approved by the Institutional Bioethics and Biosecurity Committee of the Universidad Austral de Chile (Protocol Nº 56 and 332) and conducted according to NIH Guidelines for the use of animals in research. For leukocyte adhesion, we used 5 animals in each experimental group. Briefly, Rockefeller male mice (2–3 months old) were anesthetized with Ketamine-Xylazine mixture ip (K, 100 mg/kg; X, 10 mg/kg). The cremaster was gently exteriorized through a midline scrotum incision and visualized with an inverted microscope (objective: × 32, 0.4 N.A.) equipped with a TV camera (Hamamatsu, Middlesex, NJ) connected to the microscope. IL-8 or MDA-CM was topically added to the cremaster and leukocytes adhering to venules were filmed for 30 min while a phosphate-buffered saline solution was applied topically. One venule was recorded per animal. In the experiments designed to assess the role of NO, l-NMA was administered at 50 mg/Kg for 30 min via the caudal tail vein before the stimulation with IL-8 or MDA-CM. At the end of the experiment, animals were euthanized by an anesthetic overdose with Ketamine–Xylazine mixture ip. For lung metastasis studies, we followed protocols previously described (Bailey-Downs et al. 7). 8–10 week-old BALB/c female mice were injected orthotopically into the four mammary fat pad with 2 × 10^5^ 4T1 cells. After primary tumor formation (4 days), mice were divided in two groups: (1) control injected daily with PBS and (2) injected daily with NAC (200 mg/kg). After 28 days, primary tumors were dissected from the surrounding tissues, weighed, and measured with calipers. Thickness, width, and length of the tumor were recorded. Lungs were extracted and superficial metastases were counted.

### Histopathological analysis

Tumor and metastasis samples obtained from animals were embedded in paraffin wax and cut in 10 µm sections for hematoxylin–eosin staining. For lung immunohistochemistry, 5 μm sections were cut, dewaxed, and rehydrated. Sections were treated with absolute methanol/3% H_2_O_2_ for 15 min to quench endogenous pseudoperoxidase activity, rinsed in distilled water and then in Tris–HCl buffer. After blocking non-specific binding for 20 min with horse serum (Vecton, Carpinteria, CA, USA), tissue sections were incubated with anti-*S*-Nitroso-Cysteine. The antibody was reconstituted and diluted in 0.01 M PBS containing 1% IgG-free bovine serum albumin (BSA; Sigma-Aldrich, St. Louis, MO, USA) and 0.02% NaN_3_. Bound antibodies were detected using the R.T.U VECTASTAIN Detection Kit (Vector Laboratories) and peroxidase was visualized with Liquid-3,3ʹdiaminobenzidine (DAB) + Substrate Chromogen System (DAKO). Slides were counterstained with hematoxylin–eosin and mounted with mounting medium (DAKO). Cells were visualized using a light microscope (Zeiss).

### Statistical analysis

Experiments were conducted in groups with a minimum of 5 replicates (n = 5). Data are expressed as mean ± standard error. Apparent differences were assessed for statistical significance using GraphPad Prism and Sigma Plot software. Significance was accepted at p < 0.05. Details of the specific statistical analysis are indicated in each figure.

## Data Availability

The datasets used and/or analysed during the current study are available from the corresponding author on reasonable request.

## Abbreviations

IL-8: Interleukin-8
NO: Nitric oxide
eNOS: Endothelial nitric oxide synthase
l-NMA: *NG*-Methyl-l-arginine
SNO: *S*-Nitrosylated
VCAM-1: Vascular adhesion molecule-1
GC-1: Soluble guanylyl cyclase
NAC: *N*-Acetyl-cysteine
